# Artificial Intelligence-Derived Fractional Flow Reserve in Routine Clinical Practice: An International Multicenter Retrospective Study

**DOI:** 10.1016/j.jscai.2026.105451

**Published:** 2026-06-30

**Authors:** Eyal Ben-Assa, Bruce A. Samuels, Ehtisham Mahmud, Carlos Cafri, Ariel Roguin, Yair Feld, Eli Lev, Emanuel Harari, Tali Ben-Yehuda, Hector M. Garcia-Garcia, Gregg W. Stone

**Affiliations:** a Assuta Ashdod University Hospital, Ben-Gurion University of the Negev, Ashdod, Israel; b Institute for Medical Engineering and Science, Massachusetts Institute of Technology, Cambridge, Massachusetts; c Cedars-Sinai Medical Center, Los Angeles, California; d Sulpizio Cardiovascular Institute, University of California, San Diego, La Jolla, California; e Soroka Medical Center, Ben-Gurion University of the Negev, Beer Sheva, Israel; f Hillel Yaffe Medical Center, Hadera, Ruth and Bruce Rappaport Faculty of Medicine, Technion Institute of Technology, Haifa, Israel; g Tzafon (Poriya) Medical Center, Azrieli Faculty of Medicine, Bar Ilan University, Tveria, Israel; h MedStar Washington Hospital Center, Washington, DC; i Icahn School of Medicine at Mount Sinai, New York, New York

**Keywords:** angiography, artificial intelligence, artificial intelligence-derived fractional flow reserve, fractional flow reserve

## Abstract

**Background:**

Artificial intelligence-based fractional flow reserve (AI-FFR) incorporates a machine learning-based algorithm to derive FFR directly from angiography. Its accuracy compared with invasive FFR has not been assessed.

**Methods:**

AI-FFR was compared with wire-based FFR in patients with a single intermediate lesion (diameter stenosis ≥40% to <70%) at 5 centers in the United States and Israel. AI-FFR assessments were performed by core laboratory analysts blinded to invasive FFR results. Diagnostic performance metrics were calculated using an FFR threshold of ≤0.80.

**Results:**

A total of 504 vessels from 496 patients were analyzed. AI-FFR computation time was 36.1 ± 7.7 seconds. Lesion detection was fully automatic in 371 of 504 (73.6%) vessels, whereas semiautomated analysis with manual marking was performed in 133 of 504 (26.4%) vessels. Mean wire-based FFR was 0.85 ± 0.07 and mean AI-FFR was 0.85 ± 0.08 (mean difference, 0.00 ± 0.08; 95% CI, −0.15 to 0.15; *P* = .41). AI-FFR showed a sensitivity of 90.2%, specificity of 94.9%, positive predictive value of 83.5%, negative predictive value of 97.1%, and overall diagnostic accuracy of 93.8%. The area under the receiver operating characteristic curve (AUC) was 0.93 (95% CI, 0.89-0.96). Among 151 lesions with wire-based FFR values in the borderline “gray zone” (0.75-0.85), AI-FFR demonstrated a diagnostic accuracy of 91.4% and an AUC of 0.91 (95% CI, 0.86-0.96). AI-FFR demonstrated high diagnostic accuracy across vessel types and lesion locations in men and women and between the US and Israeli cohorts.

**Conclusions:**

AI-FFR, an automated, machine learning-based tool, demonstrated high diagnostic accuracy compared with wire-based FFR. Its speed, simplicity, and independence from complex procedural steps may facilitate broader adoption during coronary angiography.

## Introduction

Physiological assessment is crucial for evaluating coronary artery lesions and guiding revascularization decisions. The use of fractional flow reserve (FFR) has consistently demonstrated improvements in clinical outcomes and is endorsed by major cardiovascular guidelines.^1^, ^2^, ^3^, ^4^ Despite its proven benefits, the routine use of FFR is hampered by extended procedure times, risks of invasive placement of coronary wires and side effects from hyperemic agents, and increased costs. As a result, real-world data consistently show that FFR remains significantly underutilized in clinical practice.^5^ These challenges have driven the pursuit of safer and faster alternatives, such as minimally-invasive angiographic-based computational physiological assessment.^6^, ^7^, ^8^ However, these techniques still face limitations due to procedural complexity and lengthy processing times.^9^

Over the past decade, several angiography-based physiological tools that rely on computation-based flow models have been developed and validated against invasive FFR. These include quantitative flow ratio (QFR), vessel FFR, and angiography-derived FFR, which have demonstrated good diagnostic performance but are limited by technical requirements, acquisition constraints, and moderate reproducibility in real-world settings.^10^ In a recent randomized trial, QFR-guided revascularization was associated with higher event rates and more interventions compared with an FFR-guided strategy, highlighting the critical importance of precision in flow estimation.^11^

Recently, advancements in artificial intelligence (AI) have led to the development of AI-derived FFR (AI-FFR),^9^^,^^12^^,^^13^ a novel approach that leverages machine learning algorithms to analyze coronary angiograms and generate vessel-specific FFR values. Unlike other angiography-based technologies that rely on computation-based flow models that often require specific image acquisition protocols, manual lesion annotation, and hemodynamic inputs, AI-FFR is entirely based on deep learning. Trained on tens of thousands of annotated angiographic frames paired with invasive FFR data, the algorithm autonomously extracts vessel anatomy from a minimum of 2 views, detects stenoses, and calculates FFR through a purely data-driven process. The result is an automated system that delivers rapid FFR estimation in <1 minute, with minimal operator input and no need for hyperemia or acquisition constraints. By eliminating many of the limitations associated with current technologies, AI-FFR offers a streamlined approach to functional assessment during routine coronary angiography.

In an initial feasibility study conducted in Israel,^12^ AI-FFR demonstrated high diagnostic accuracy (area under the receiver operating characteristic curve [AUC] 0.93), rapid analysis times (∼37 seconds), and required minimal operator input. To further validate its performance in multiple geographies and across varying angiographic systems, acquisition protocols, and pressure-wire platforms, we conducted an international, multicenter, retrospective study including additional patients from Israel and the United States.

## Methods

### Study design and population

The present international, multicenter, retrospective study was performed to evaluate the diagnostic performance of a machine learning-derived angiographic FFR (AI-FFR) tool—AutocathFFR (MedHub-AI)—compared with invasive pressure-wire–based FFR measurements. The study included 5 clinical centers: 3 in Israel (Rambam Health Care Campus, Haifa; Hillel Yaffe Medical Center, Hadera; Soroka Medical Center, Be’er Sheva) and 2 in the United States (MedStar Washington Hospital Center, Washington, DC; and UC San Diego Health, San Diego, California). The study population comprised patients who underwent invasive coronary angiography and had FFR measured in at least 1 coronary artery between September 2019 and June 2023.

All consecutive patients undergoing invasive coronary angiography with FFR assessment at the participating centers were screened retrospectively. Cases were included if they the met the following predefined criteria: age >18 years; stable angina or non–ST-elevation acute coronary syndrome (unstable angina or non-ST-segment elevation myocardial infarction); left ventricular ejection fraction ≥30%; and with a single intermediate lesion (diameter stenosis ≥40% to <70% by site visual assessment) in at least one nonculprit coronary artery assessed by invasive FFR. There were no specific lesion length or reference vessel diameter requirements for study lesions. Patients with conditions or anatomies for which invasive FFR is not validated—such as severe tortuosity, heavy calcification, ostial lesions, prior coronary artery bypass graft, or tandem/diffuse disease—were excluded. The study was approved by the institutional review boards at all participating centers, and the protocol was registered at ClinicalTrials.gov (NCT04861519).

### Invasive coronary angiography and wire-based FFR acquisition

Coronary angiography was performed using standard techniques, with cine acquisition at ≥7 frames per second. For each lesion, at least 2 angiographic projections were required, although no specific angulation was mandated.

Invasive FFR was performed according to clinical guidelines. Hyperemia was induced using either intravenous adenosine (140-180 μg/kg/min) or an intracoronary adenosine bolus (100 μg for the right coronary artery, 200 μg for the left coronary artery), per operator preference. The pressure wire was equalized at the ostium then advanced ≥20 mm distal to the target lesion. Pressure tracings were stored and centrally evaluated for waveform quality and pressure-wire drift. Pressure-wire systems included Abbott (St. Jude), Philips, and Opsens.

### Artificial intelligence-based FFR software and algorithm description

Artificial intelligence-based FFR values are generated using the AutocathFFR software. AI-FFR computation is performed using a multistage deep learning pipeline applied directly to routine Digital Imaging and Communications in Medicine (DICOM) angiographic cine runs. After automatic ingestion of the cine sequence, preprocessing includes temporal alignment, frame normalization, and quality assessment. A convolutional neural network-based segmentation model extracts coronary vessel contours and centerlines across frames. Temporal tracking algorithms maintain vessel continuity throughout the cardiac cycle. Automated frame selection is performed using image quality metrics that assess contrast opacification, vessel sharpness, motion stability, and overlap detection. The system identifies frames with optimal lesion visualization; continuous contrast filling over 2 complete cardiac cycles is not required. For each selected projection, the model extracts >150 spatial and temporal features per frame, including geometric characteristics, lesion morphology, contrast dynamics, and gradients (eg, curvature profiles, luminance gradients, lesion length, vessel tapering). A multiview fusion network integrates information across projections and cardiac phases. The final AI-FFR estimate is generated using a supervised deep regression model trained on angiographic data paired with invasive FFR values as reference labels. The system was trained on >13,000 coronary angiographic studies, >10,000 manually labeled still-frame images, and >1500 vessels with matched invasive FFR values serving as ground-truth labels. The software automatically detects and analyzes stenoses, providing per-lesion–AI-FFR estimates in <60 seconds. If necessary, manual lesion selection may be performed.

The AutocathFFR system is fully integrated into the cardiac catheterization laboratory workflow. Angiographic images are automatically streamed to a dedicated computer in the control room on which the AutocathFFR software is installed (Central Illustration). The analysis is performed, and results are displayed in real time on both the control room workstation and on the angiographic boom monitor in the procedure room, allowing immediate operator interpretation. If desired, the technician or operator can manually adjust or mark a lesion on the software interface, affording real-time modification of the AI-FFR calculation. A single AutocathFFR computer can be connected to multiple catheterization laboratories simultaneously, supporting multilaboratory integration.

**Central Illustration:**
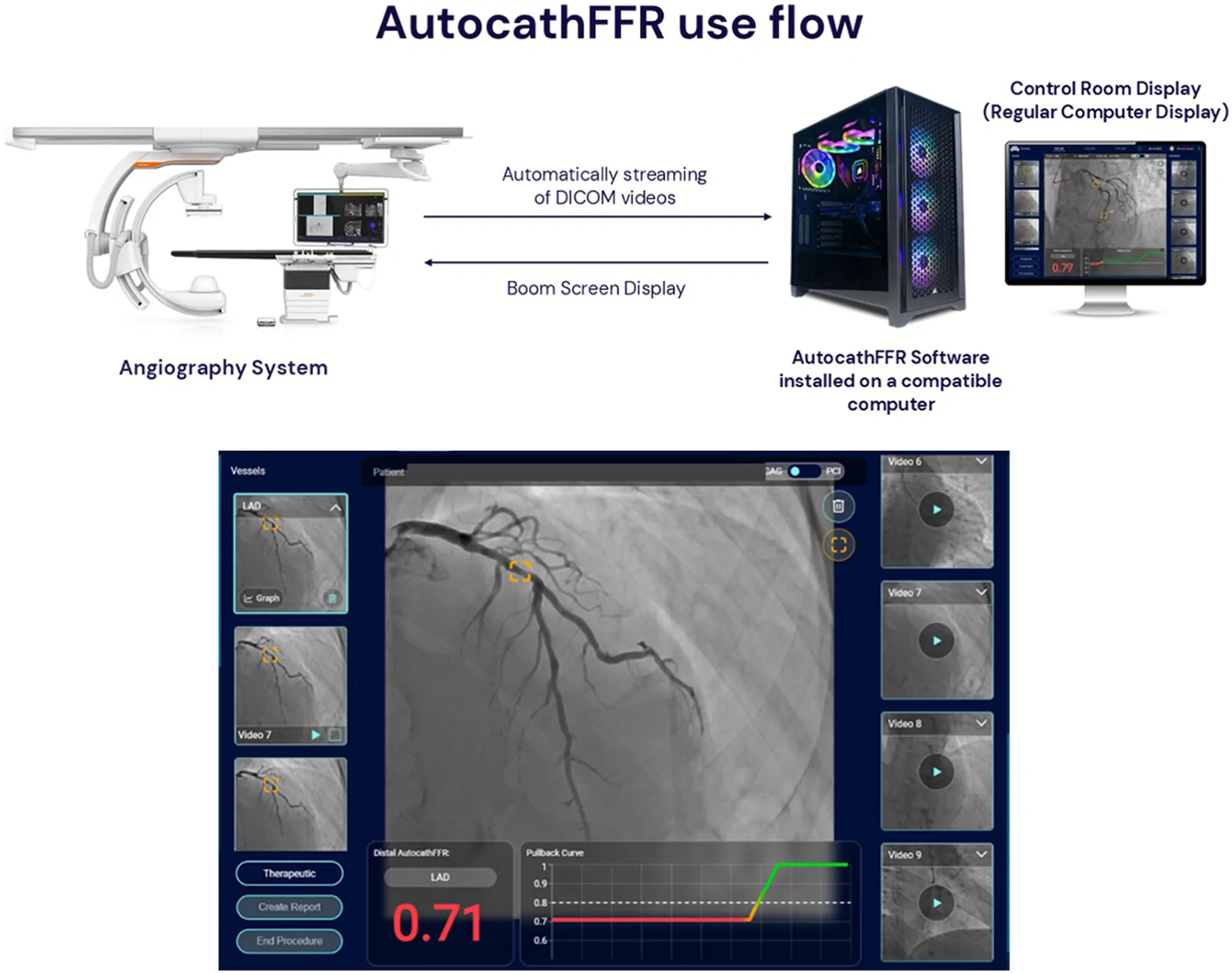
**AutocathFFRworkflow diagram and software display.** (Top) AutocathFFR System Integration Diagram: Illustration of the streamlined data flow within the catheterization laboratory. DICOM video files are automatically streamed from the angiography system to a dedicated computer where the AutocathFFR software is installed. After automated analysis, results are transmitted back and displayed simultaneously on the angiography system boom screen and the control room monitor. (Bottom) AutocathFFR Display Interface: Example of the AI-generated FFR output, showing the user interface with angiographic views, lesion markings from multiple projections, numerical AI-FFR value, visual overlay, and pullback curve simulation for the selected vessel and lesion. AI, artificial intelligence; DICOM, Digital Imaging and Communications in Medicine; FFR, fractional flow reserve; LAD, left anterior descending artery.

### Core laboratory analysis

For the present study, AI-FFR values were assessed offline at an independent central core laboratory (MedStar Cardiovascular Research Network). The core laboratory first verified angiographic image quality and FFR trace integrity according to predefined inclusion criteria. Quantitative coronary angiography was then performed using CAAS Workstation 8.1 (Pie Medical Imaging). Without knowledge of the FFR values and prior to assessing AI-FFR, the analysts then chose a discrete lesion in a vessel that had minimal overlap and foreshortening in 2 projections and sufficient image quality for reliable vessel segmentation. The automated AutocathFFR software was then used for AI-based FFR analysis, including manual lesion annotation when required.

### Statistical analysis

Artificial intelligence-based FFR and invasive FFR were compared using Pearson correlation, Bland–Altman analysis, and receiver operating characteristic curves. Diagnostic performance was assessed by calculating sensitivity, specificity, positive and negative predictive values, overall accuracy, and AUC by Youden method using a cutoff of 0.80. Paired *t* tests and Kolmogorov–Smirnov tests were used as appropriate. Subgroup analyses by vessel type (left anterior descending artery [LAD], right coronary artery [RCA], left circumplex artery [LCX]), lesion location (proximal, mid, distal), sex, and geographic region (United States, Israel) were also performed. Statistical analyses were conducted using SPSS Statistics 25 (IBM Corp).

## Results

### Study population and vessels

A total of 496 patients were included in the present analysis; 297 were previously reported in a single-country retrospective study,^12^ whereas 199 represent a new multinational cohort from 2 US centers. A total of 504 vessels with a single intermediate lesion were analyzed in 496 patients at the 5 centers, including 297 patients from Israel and 199 patients from the United States. Overall, 339 of the 496 patients (68.3%) were men, 205 of 496 (41.3%) had diabetes mellitus, and 131 of 297 (44.1%) presented with non–ST-elevation acute coronary syndrome (Israeli data only). The most frequently involved vessel was the LAD, representing 376 of 504 (74.6%) of all lesions. By quantitative coronary angiography, the mean angiographic stenosis severity of the intermediate lesion was 50.8% ± 9.6%, the mean lesion length was 13.8 ± 6.6 mm, and the mean reference vessel diameter was 2.74 ± 0.59 mm (Table 1).

**Table 1:** Baseline characteristics – patients and lesions

Patients	N = 496
Age, y	64.8 ± 10.1
Male sex	339/496 (68.3)
Body mass index, kg/m^2^	29.2 ± 5.5
Diabetes mellitus	205/496 (41.3)
Previous PCI	166/496 (33.5)
Current smoker	147/496 (29.6)
NSTE-ACS^a^	131/297 (44.1)

Lesions (QCA)	N = 504

Vessel location	
Left anterior descending artery	376/504 (74.6)
Left circumflex artery	71/504 (14.1)
Right coronary artery	57/504 (11.3)
Vessel segment	
Proximal	169/504 (33.5)
Mid	262/504 (52.0)
Distal	48/504 (9.5)
Reference vessel diameter^a^, mm	2.74 ± 0.59
Diameter stenosis, %	50.8 ± 9.8
Lesion length, mm	13.8 ± 6.6
Calcification, moderate or severe	119/504 (23.6)
Bifurcation	190/504 (37.7)

Values of continuous variables are reported as mean ± SD and those of categorical variables as n/N (%).

NSTE-ACS, non-ST-segment elevation acute coronary syndrome; PCI, percutaneous coronary intervention; QCA, quantitative coronary angiography.

a Israeli data only.

### Invasive wire-based FFR

The mean wire-based FFR was 0.85 ± 0.07, and 112 of 504 (22.2%) of FFR values were ≤0.80. The FFR value was in the gray zone (0.75-0.85) in 151 of 504 (30.0%) of cases.

### Artificial intelligence-based FFR determination

The mean processing time per case was 36.1 ± 7.7 seconds. Completely automatic lesion detection and AI-FFR computation of the desired lesions occurred in 371 of 504 (73.6%) vessels. Semiautomated analysis with manual lesion marking was performed in the remaining 133 of 504 (26.4%) vessels.

### Agreement between AI-FFR and invasive FFR

**Figure 1:**
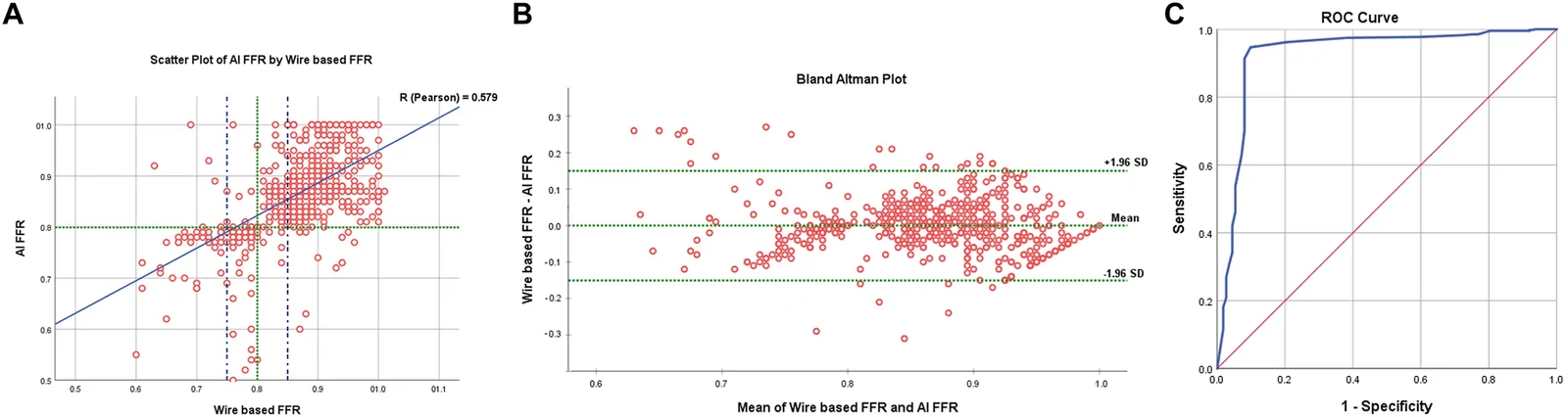
**Relationship between invasive FFR and AI-FFR.** (A) Scatterplot showing the relationship between AI-derived FFR and invasive FFR measurements across all analyzed vessels (N = 504). The green dotted horizontal and vertical lines indicate the diagnostic threshold of 0.80 for AI-FFR and wire-based FFR, respectively. Dashed vertical lines represent the gray zone FFR range (0.75-0.85). (B) Bland–Altman plot demonstrating the agreement between AI-FFR and invasive FFR, with solid lines representing the mean difference and ±2 standard deviations. (C) Receiver operating characteristic (ROC) curve for AI-FFR, using invasive FFR as the reference standard. Area under the curve = 0.93. AI, artificial intelligence; FFR, fractional flow reserve.

As shown in Table 2, in the 504 vessels the mean AI-FFR measured 0.85 ± 0.08. The mean difference between invasive FFR and AI-FFR was 0.00 ± 0.08 (95% CI, −0.15 to 0.15; *P* = .41). There was moderate correlation between the 2 measurements (Pearson *R* = 0.59; *P* < .001), with a regression slope of 0.647 (95% CI, 0.569-0.724), an intercept of 0.305 (95% CI, 0.167-0.442), and an intraclass correlation coefficient of 0.305 (95% CI, 0.167-0.442) (Figure 1A). A Bland–Altman plot demonstrated good agreement between modalities, without major systematic bias (Figure 1B).

**Table 2:** Comparison of wire-based FFR and AI-FFR

	Total population	Gray zone analysis	
		In the gray zone	Outside the gray zone
		Wire-based FFR 0.75-0.85	Wire-based FFR <0.75 and >0.85
	N = 504 vessels	n = 151 vessels	n = 353 vessels
Wire-based FFR	0.85 ± 0.07	0.81 ± 0.03	0.89 ± 0.08
AI-FFR	0.85 ± 0.08	0.82 ± 0.09	0.88 ± 0.08
Difference	0.00 ± 0.08	−0.01 ± 0.08	0.00 ± 0.08
*P* value	.41	.13	.25
Sensitivity	90.2%	89.9%	90.7%
Specificity	94.9%	92.7%	95.5%
PPV	83.5%	91.2%	73.6%
NPV	97.1%	91.6%	98.7%
Accuracy	93.8%	91.4%	94.9%
AUC (95% CI)	0.93 (0.89-0.96)	0.91 (0.86-0.96)	0.93 (0.88-0.98)

AI, artificial intelligence; AUC, area under the curve; FFR, fractional flow reserve; NPV, negative predictive value; PPV, positive predictive value.

Using a diagnostic threshold of ≤0.80 for wire-based FFR, AI-FFR yielded a sensitivity of 90.2% (95% CI, 83.1-95.0), specificity of 94.9% (95% CI, 92.2-96.9), positive predictive value (PPV) of 83.5%, negative predictive value (NPV) of 97.1%, and overall diagnostic accuracy of 93.8% (Table 2). The AUC was 0.93 (95% CI, 0.89-0.96) (Figure 1C).

### Gray zone performance

Among the 151 lesions with invasive FFR values within the borderline "gray zone" range (0.75-0.85), the mean invasive FFR was 0.81 ± 0.03 and the mean AI-FFR was 0.82 ± 0.09 (mean difference, −0.01 ± 0.08; 95% CI, −0.03 to 0.01; *P* = .13) (Table 2). Sensitivity was 89.9%, specificity was 92.7%, PPV was 91.2%, NPV was 91.6%, overall diagnostic accuracy was 91.4%, and the AUC was 0.91 (95% CI, 0.86-0.96). Among the 353 lesions outside the gray zone (invasive FFR <0.75 or >0.85), the mean invasive FFR was 0.89 ± 0.08 and the mean AI-FFR was 0.88 ± 0.08 (mean difference, 0.00 ± 0.08; 95% CI, 0.00 to 0.01; *P* = .25) (Table 2). Sensitivity was 90.7%, specificity was 95.5%, PPV was 73.6%, NPV was 98.7%, overall diagnostic accuracy was 94.9%, and the AUC was 0.93 (95% CI, 0.88-0.98).

### Analysis in subgroups

Artificial intelligence-based FFR demonstrated consistent and high diagnostic accuracy across the 3 major coronary arteries. Sensitivity was 89.8% in LAD lesions, 100% in RCA, and 88.9% in LCX, whereas specificity exceeded 94% in all 3 territories (Table 3). The AUC was ≥0.92 across all vessel types, perhaps somewhat better in the RCA (0.97) than in the LAD and LCX (0.92 in each). Logistic regression analysis using vessel type as a covariate (LAD as reference) found no statistically significant interaction affecting AI-FFR diagnostic performance across vessels (*P* = .97 for LCX; *P* = .99 for RCA), suggesting comparable sensitivity and specificity across the 3 epicardial coronary vessels. Diagnostic accuracy was also high in vessels with lesions in the proximal, mid, and distal coronary segments (Table 3).

**Table 3:** Comparison of wire-based FFR and AI-FFR in subgroup 1

	Vessel type			Lesion location in vessel		
	LAD	LCX	RCA	Proximal	Mid	Distal
	n = 376 vessels	n = 71 vessels	n = 57 vessels	n = 169 vessels	n = 286 vessels	n = 49 vessels
Wire-based FFR	0.85 ± 0.07	0.90 ± 0.08	0.90 ± 0.07	0.86 ± 0.08	0.86 ± 0.08	0.85 ± 0.10
AI-FFR	0.85 ± 0.08	0.89 ± 0.09	0.89 ± 0.08	0.86 ± 0.09	0.86 ± 0.07	0.85 ± 0.08
Difference	0.00 ± 0.08	0.01 ± 0.09	0.01 ± 0.08	0.00 ± 0.07	0.00 ± 0.08	0.00 ± 0.09
*P* value	.41	.29	.26	.78	.80	.70
Sensitivity	89.8%	88.9%	100.0%	87.5%	91.4%	92.9%
Specificity	95.0%	95.2%	94.2%	95.3%	93.9%	97.2%
PPV	86.3%	72.7%	63.5%	85.4%	79.1%	100%
NPV	96.4%	98.3%	100.0%	96.1%	97.7%	100.0%
Accuracy	93.6%	94.4%	94.7%	93.5%	93.4%	98.0%
AUC (95% CI)	0.92 (0.87-0.96)	0.92 (0.79-1.00)	0.97 (0.93-1.00)	0.91 (0.85-0.98)	0.93 (0.88-0.97)	0.96 (0.88-1.00)

AI, artificial intelligence; AUC, area under the curve; FFR, fractional flow reserve; LAD, left anterior descending artery; LCX, left circumplex artery; NPV, negative predictive value; PPV, positive predictive value; RCA, right coronary artery.

Among 157 women in whom analysis of 160 vessels with intermediate lesions was performed, mean invasive FFR was 0.88 ± 0.07 and AI-FFR was 0.89 ± 0.08 (*P* = .25); AI-FFR had sensitivity of 100%, specificity of 97.1%, PPV of 84.6%, NPV of 100%, and overall diagnostic accuracy of 97.5% (Table 4). Among 339 men with 344 vessels with intermediate lesions, invasive FFR was 0.85 ± 0.08 and AI-FFR was 0.85 ± 0.08 (*P* = .39). AI-FFR had sensitivity of 87.8%, specificity of 93.7%, PPV of 83.2%, NPV of 95.6%, and overall accuracy of 92.2% (Table 4). Logistic regression analysis using sex as a covariate found no statistically significant interaction with AI-FFR diagnostic performance (*P* = .99 for sensitivity; *P* = .15 for specificity).

**Table 4:** Comparison of wire-based FFR and AI-FFR in subgroup 2.

	Sex		Geographic region	
	Female	Male	United States	Israel
	n = 160 vessels	n = 344 vessels	n = 200 vessels	n = 304 vessels
Wire-based FFR	0.88 ± 0.07	0.85 ± 0.08	0.87 ± 0.08	0.85 ± 0.07
AI-FFR	0.89 ± 0.08	0.85 ± 0.08	0.88 ± 0.11	0.85 ± 0.06
Difference	−0.01 ± 0.07	0.00 ± 0.08	−0.01 ± 0.09	0.01 ± 0.07
*P* value	.25	.39	.29	.16
Sensitivity	100.0%	87.8%	88.4%	91.3%
Specificity	97.1%	93.7%	95.5%	94.5%
PPV	84.6%	83.2%	84.4%	82.9%
NPV	100.0%	95.6%	96.8%	97.4%
Accuracy	97.5%	92.2%	94.0%	93.8%
AUC (95% CI)	0.97 (0.93–1.00)	0.97 (0.93–1.00)	0.92 (0.85–0.98)	0.93 (0.89–0.97)

NSTE-ACS, non-ST-segment elevation acute coronary syndrome. Other abbreviations as in Table 2.

AI, artificial intelligence; AUC, area under the curve; FFR, fractional flow reserve; NPV, negative predictive value; PPV, positive predictive value.

Finally, diagnostic accuracy of AI-FFR compared with invasive FFR was consistent in patients enrolled in the United States and Israel (Table 4).

## Discussion

In this retrospective, international, multicenter study, we evaluated the diagnostic performance of an automated AI-based software (AutocathFFR) that calculates AI-FFR values directly from coronary angiograms without the need for pressure wires, adenosine, or specific angiographic acquisition. The principal findings are that AI-FFR demonstrated high diagnostic accuracy compared with invasive wire-based FFR across a wide range of patients and lesions.

The present study included 504 vessels that contained an angiographic intermediate lesion from 496 patients across 5 centers in Israel and the United States, with core laboratory analysis conducted independently. To date, this is the largest study of AI-FFR conducted to evaluate the AutocathFFR algorithm. Previous studies have assessed its performance in smaller cohorts, including a feasibility study of 31 patients^13^ and a 297-patient retrospective analysis from Israel.^12^ The present study builds upon those findings by incorporating an additional 199 patients from 2 US centers, significantly enhancing the geographic diversity and generalizability of the analysis.

Our findings suggest that AI-FFR can provide rapid and accurate physiological assessment of intermediate coronary lesions, achieving a sensitivity of 90%, specificity of 95%, overall diagnostic accuracy of 94%, and an AUC of 0.93 compared with invasive FFR. These results were consistent across major subgroups, including vessel type and lesion location, sex, and enrollment in the United States or Israel. Calculation time was brief at 36 seconds, with 74% of cases performed fully automatically. Importantly, its diagnostic performance remained robust in the clinical gray zone (invasive FFR 0.75-0.85), with an AUC of 0.91 and accuracy of 91%, a range where physiological accuracy is most critical, underscoring the robustness of AI-FFR as a clinically reliable tool. Although this high accuracy provides confidence to use AI-FFR for clinical decision making within the gray zone, AI-FFR, like invasive FFR, is only a single test, and the true gold standard for ischemia is elusive. Thus, if the AI-FFR result in an individual case provides contrary direction to that suspected clinically, use of wire-based FFR or other testing may be helpful for further evaluation.

Our study demonstrated a significant yet moderate overall correlation coefficient (*R* = 0.59) between AI-FFR and invasive FFR. This may reflect reduced model precision at physiological extremes, which were underrepresented in the training dataset. However, at these extremes—very high or very low FFR values—treatment decisions are often straightforward, and diagnostic accuracy of AI-FFR was especially high (94.9%).

The novel methodology by which AI-FFR determines vessel physiology varies substantially from other angiography-based systems. Unlike existing approaches that rely on flow-based estimations such as from computational fluid dynamics or resistive network modeling, AI-FFR utilizes an automated, machine learning-based experience to derive FFR values from standard angiograms without requiring pressure wires, specific projections, or user annotations. Whereas most currently available methods integrate AI for segmentation or workflow support,^9^ AI-FFR uniquely employs AI and machine learning as the core mechanism for FFR estimation, enabling both lesion detection and physiological calculation with minimal operator input.

These distinctions have practical implications. Computational models often require specific acquisition angles (most commonly from multiple projections) and prohibit table panning, necessitating manual annotations and multiple steps of user input. For example, angiography-derived FFR requires 3 angiographic views and has a reported analysis time of 4.3 ± 3.4 minutes per lesion,^9^ whereas the average analysis time for 2-view QFR was 3.9 ± 1.4 minutes per lesion in the FAVOR III China trial.^14^ In contrast, AutocathFFR analyzes standard coronary angiograms without acquisition constraints, performs fully automated lesion detection in ∼75% of cases, and delivers results in <40 seconds. This automation improves workflow efficiency, reduces training burden, and allows retrospective offline analysis—even on angiograms from other institutions.

For an angiography-derived physiology system to be useful, it must not only be rapid but also provide high diagnostic accuracy, especially in the borderline gray zone of invasive FFR values where appropriate clinical decision making demands precision. Recent studies underscore this issue. In 2023, a blinded analysis comparing 5 computational angiography-derived FFR methods reported only moderate diagnostic accuracy for each, with notable false-positive rates in larger vessels and in invasive FFR gray zone lesions (between 0.75 to 0.85).^10^ Similarly, the FAVOR III Europe trial failed to demonstrate noninferiority of QFR-guided percutaneous coronary intervention compared with invasive FFR, driven by higher rates of periprocedural myocardial infarction and major adverse cardiac events arising late from QFR-deferred lesions.^11^

In the present study, the diagnostic performance of AI-FFR was high in FFR-defined gray zone lesions, with a diagnostic accuracy of 91.4% and AUC of 0.91 compared with invasive FFR, only slightly lower than for non–gray zone lesions, 94.9% and 0.93 respectively. AI-FFR, by maintaining both automation and diagnostic precision within and outside the gray zone, may support safer, faster, and more effective decision making in the catheterization laboratory without the need for invasive pressure wires in most cases.

### Limitations

The main limitation of our study is its retrospective nature, with inherent potential for selection bias and unmeasured confounders. In particular, the meticulousness with which the invasive wire-based FFR technique was performed (eg, wire pullback to exclude drift) could not be verified. Prospective invasive FFR collection may have ensured absence of drift and other artifacts, potentially further enhancing comparative AI-FFR accuracy. Future studies in real-time clinical settings performed by multiple operators are warranted to further assess reproducibility, workflow integration, and procedural timing. Second, the relatively low prevalence of lesions with abnormal FFR (22.2%) and clustering of values near the clinical decision threshold may influence correlation metrics; further evaluation in populations with a broader physiological spectrum is warranted. Third, AI-FFR analyses in this study were performed offline in a core laboratory rather than in real time in the catheterization laboratory. Given the automated nature of the technology, although the results would have been identical in the 73.6% of cases in which user input was not required, whether the technician and interventional cardiologist would have differed in their use of manual lesion selection and the effect this might have had on the correlation with invasive FFR are unknown. Finally, exclusion criteria were similar to those from prior angiography-derived physiology studies, omitting angiographic scenarios in which FFR has not been validated, such as tandem lesions and diffuse coronary disease. Additional validation of AI-FFR is required in these scenarios. Nonetheless, this study supports the feasibility and reliability of AI-FFR in real-world settings and sets the stage for future prospective studies in more complex lesion subsets.

### Conclusions

The present international, multicenter retrospective study demonstrates that AI-FFR—an automated, machine learning-based FFR tool—can achieve high diagnostic accuracy compared with invasive FFR, both within and outside the critical gray zone. Its rapid analysis time, minimal procedural requirements, and operator independence in most cases may support broader adoption of physiologic assessment during routine coronary angiography. Prospective studies are needed to evaluate its utility in guiding revascularization decisions and improving patient outcomes.

## CRediT authorship contribution statement

**Eyal Ben-Assa:** Conceptualization, Data curation, Formal analysis, Funding acquisition, Investigation, Methodology, Project administration, Writing – original draft. **Bruce A. Samuels:** Investigation, Methodology, Writing – review & editing. **Ehtisham Mahmud:** Investigation, Writing – review & editing. **Carlos Cafri:** Data curation, Investigation, Writing – review & editing. **Ariel Roguin:** Data curation, Investigation, Writing – review & editing. **Yair Feld:** Data curation, Writing – review & editing. **Eli Lev:** Data curation, Investigation, Writing – review & editing. **Emanuel Harari:** Data curation, Writing – review & editing. **Tali Ben-Yehuda:** Data curation, Writing – review & editing. **Hector M. Garcia-Garcia:** Formal analysis, Investigation, Writing – review & editing. **Gregg W. Stone:** Conceptualization, Investigation, Methodology, Project administration, Supervision, Writing – review & editing.

## Declaration of competing interest

Eyal Ben-Assa, Yair Feld, Ariel Roguin, and Bruce Samuels are consultants for MedHub-AI. Ehtisham Mahmud has received an institutional research grant from MedHub-AI. Hector M. Garcia-Garcia has received institutional grant support from Biotronik, Boston Scientific, Medtronic, Abbott, Neovasc, Shockwave Medical, Philips, and CorFlow. Gregg W. Stone has received speaker honoraria from Boehringer Ingelheim; has served as a consultant to Millennia Biopharma, Apollo Therapeutics, Cardiac Success, Occlutech, Oxitope, Elixir, Impulse Dynamics, Asceneuron, Myochron, Vesalio, HeartFlow, Colibri, Bioventrix, Abbott, Remote Cardiac Enablement, Valfix, Zoll, Shockwave Medical, Adona Medical, HighLife, Elucid Bio, Aria, Alleviant, FBR Medical, MedHub, Biotyx Medical, and Ablative Solutions; and has equity/options from Cardiac Success, Ancora, Cagent, Applied Therapeutics, Biostar family of funds, SpectraWave, Orchestra Biomed, Aria, Valfix, Xenter, Vascentis. Gregg W. Stone’s employer, Mount Sinai Hospital, receives research grants from Shockwave Medical, Biosense-Webster, Bioventrix, Abbott, Abiomed, Cardiovascular Systems Inc, Phillips, Pulnovo, V-wave, and PCORI (via Weill Cornell Medical Center). Ehtisham Mahmud, Carlos Cafri, Eli Lev, Emanuel Harari, and Tali Ben-Yehuda have no disclosures relevant to the current study to declare.
